# Integrin α3β1 Represses Reelin Expression in Breast Cancer Cells to Promote Invasion

**DOI:** 10.3390/cancers13020344

**Published:** 2021-01-19

**Authors:** Abibatou Ndoye, Rakshitha Pandulal Miskin, C. Michael DiPersio

**Affiliations:** 1 Department of Surgery, Albany Medical College, Albany, 12208 NY, USA; Ndoyea@amc.edu; 2 Department of Regenerative and Cancer Cell Biology, Albany Medical College, Albany 12208, NY, USA; Miskinr@amc.edu; 3 Department of Molecular & Cellular Physiology, Albany Medical College, Albany, 12208 NY, USA

**Keywords:** integrin α3β1, Reelin, RELN, tumor microenvironment, cancer cell secretome, invasion, triple negative breast cancer

## Abstract

**Simple Summary:**

Breast cancer remains the second leading cause of cancer-related deaths in women, and about 1 in 8 women in the United States develops invasive breast cancer in her lifetime. Integrin α3β1 has been linked to breast cancer progression, but mechanisms whereby it promotes tumor invasion remain unclear. The goal of our study was to determine how α3β1 drives invasion, towards exploiting this integrin as a therapeutic target for breast cancer. We found that α3β1 represses the expression of Reelin, a secreted glycoprotein that inhibits invasion and for which loss of expression is associated with poor prognosis in breast cancer. We also show that increased Reelin expression following RNAi-mediated suppression of α3β1 causes a significant decrease in breast cancer cell invasion. Our findings demonstrate a critical role for α3β1 in promoting cell invasion through repression of Reelin, highlighting the potential value of this integrin as a therapeutic target for breast cancer.

**Abstract:**

Integrin α3β1, a cell adhesion receptor for certain laminins, is known to promote breast tumor growth and invasion. Our previous gene microarray study showed that the RELN gene, which encodes the extracellular glycoprotein Reelin, was upregulated in α3β1-deficient (i.e., α3 knockdown) MDA-MB-231 cells. In breast cancer, reduced RELN expression is associated with increased invasion and poor prognosis. In this study we demonstrate that α3β1 represses RELN expression to enhance breast cancer cell invasion. RELN mRNA was significantly increased upon RNAi-mediated α3 knockdown in two triple-negative breast cancer cell lines, MDA-MB-231 and SUM159. Modulation of baseline Reelin levels altered invasive potential, where enhanced Reelin expression in MDA-MB-231 cells reduced invasion, while RNAi-mediated suppression of Reelin in SUM159 cells increased invasion. Moreover, treatment of α3β1-expressing MDA-MB-231 cells with culture medium that was conditioned by α3 knockdown MDA-MB-231 cells led to decreased invasion. RNAi-mediated suppression of Reelin in α3 knockdown MDA-MB-231 cells mitigated this effect of conditioned-medium, identifying secreted Reelin as an inhibitor of cell invasion. These results demonstrate a novel role for α3β1 in repressing Reelin in breast cancer cells to promote invasion, supporting this integrin as a potential therapeutic target.

## 1. Introduction

Despite significant advances in the development of therapeutic strategies, breast cancer remains the second leading cause of cancer-related deaths in women in the U.S., highlighting the need for continued research to identify molecular mechanisms that promote breast cancer progression [1]. The tumor microenvironment (TME) has become a major focus of cancer research efforts towards identifying therapeutic targets, and it includes structural proteins of the extracellular matrix (ECM), various ECM-associated proteins, and ECM-modifying enzymes, all of which may be secreted into the extracellular space by either tumor cells or stromal cells, as reviewed [2]. The ECM is composed of glycoproteins (e.g., collagens, laminins, fibronectin) and proteoglycans, which together form a complex network that maintains cellular homeostasis and normal tissue function [3,4]. Numerous studies have revealed a crucial role for the ECM in the development and progression of triple negative breast cancer (TNBC), where it plays a prominent role in neoplastic survival and cancer progression [5,6]. For example, collagen type I has been shown to induce the expression of MMP-9 in the TNBC cell line, MDA-MB-231 [7], and fibronectin and laminin were identified as potential biomarkers of metastatic TNBC [6].

Given the importance of the ECM for cancer development and progression, it is not surprising that integrin receptors for the ECM have emerged as important drivers of breast tumorigenesis and cancer progression [8,9], a role that implicates them as potential therapeutic targets. In particular, integrins that bind to certain laminins (e.g., laminins-332 and -511) have been linked to TNBC malignancy [10,11]. Integrins are heterodimeric cell surface receptors composed of an α and a β subunit, which upon binding to their extracellular ligands can signal bidirectionally to regulate a variety of essential biological processes such as cell motility, migration, survival and differentiation [12,13]. Indeed, breast cancer cells utilize the integrins that they express to promote their growth, survival, and invasiveness [8,9]. The laminin-binding integrin α3β1 has emerged as an attractive therapeutic target for breast cancer [9]. We previously showed that suppression of integrin α3β1 in the TNBC cell line, MDA-MD-231, achieved through shRNA-mediated stable knockdown of the α3 subunit, caused decreased tumor growth and invasion [14]. In a subsequent study, use of an Affymetrix gene microarray platform to assess changes in the transcriptome of α3 knockdown (α3-KD) MDA-MD-231 cells revealed numerous α3β1-regulated genes, some of which have been implicated in pro-tumorigenic cell functions [15]. Among these genes, RELN was significantly upregulated in α3-KD MDA-MB-231 cells compared with control cells, indicating α3β1-dependent repression of RELN gene expression. 

The RELN gene encodes a large, secreted glycoprotein, known as Reelin [16]. Several studies have shown that Reelin is critical for neuronal positioning, migration, and synaptic activity in the developing and adult brain [17], and some of its regulatory functions have been linked to its serine protease activity from within the ECM [18]. In neuronal cells, Reelin binds with high affinity to low-density lipoprotein receptors leading to the phosphorylation of cytosolic adaptor protein disabled-1 (Dab-1) and the inhibition of glycogen synthase kinase-3β (GSK3b) and actin remodeling [19,20]. Interestingly, Reelin has also been reported to interact with integrin α3β1 to modulate cellular adhesion and inhibit cortical neuron migration [21]. Reelin also has important functions in non-neuronal tissues, including the mammary gland [22]. In human breast cancer biopsies, Reelin is expressed in adjacent, normal breast epithelium, but it is absent from cancerous tissue [23]. Various studies have since shown that the RELN gene is epigenetically silenced in breast cancer, as well as in other cancers such as gastric and pancreatic cancer [23,24,25]. Moreover, low RELN expression in breast cancer is associated with increased cancer cell migration, positive lymph node status, and poor prognosis [23]. Together, these findings suggest a cancer-suppressive role for Reelin. However, a role for integrins in the regulation of Reelin expression has not been previously explored in breast cancer cells.

In the current study, we investigated a role for integrin α3β1-dependent repression of RELN in enhancing the invasive potential of TNBC cells. Previous studies have shown that Reelin expression is reduced in TNBC compared to hormone-positive breast cancer; TNBC primary and metastatic tumor samples express lower levels of Reelin compared to Her-2 positive tumors and metastases [26,27]. Consistently, we performed bioinformatic analysis of patient data using cBioPortal (Breast Invasive Carcinoma dataset, TCGA, PanCancer Atlas) to show that RELN mRNA is reduced in basal-like subtypes (generally considered triple-negative) compared to normal samples. Using two TNBC cell lines, MDA-MB-231 and SUM159, we show that suppression of α3β1, achieved through RNAi-mediated knockdown of the α3 integrin subunit, leads to a significant increase in RELN mRNA. Consistent with published evidence that the RELN gene is repressed or silenced in breast cancer [23], transfection with a reporter gene construct in which a minimal RELN promoter drives luciferase expression showed that RELN promoter activity was significantly higher in α3-KD MDA-MB-231 cells, compared with control cells. We demonstrate the biological significance of these findings through assessing effects of RELN gene modulation on in vitro cell invasion, where overexpression of exogenous RELN in MDA-MB-231 cells (which have a low baseline level of Reelin) significantly decreased invasion, while suppression of endogenous RELN using short-interfering RNA (siRNA) in SUM159 cells (which have a higher baseline level of Reelin) significantly increased invasion. Moreover, treatment with conditioned medium from α3-KD MDA-MB-231 cells caused a significant decrease in the invasion of parental (i.e., α3β1-expressing) MDA-MB-231 cells, and this effect was mitigated by siRNA-mediated suppression of Reelin in the α3-KD cells, demonstrating that secreted Reelin contributed to the inhibitiory effect on invasion.

Together, our findings identify a novel role for integrin α3β1 in the modulation of the cancer cell secretome through repression of the RELN gene, leading to reduced secretion of Reelin and promoting invasion of TNBC cells.

## 2. Results

### 2.1. RNAi-Mediated Suppression of Integrin α3β1 Increases RELN Gene Expression in Breast Cancer Cells

**Figure 1:**
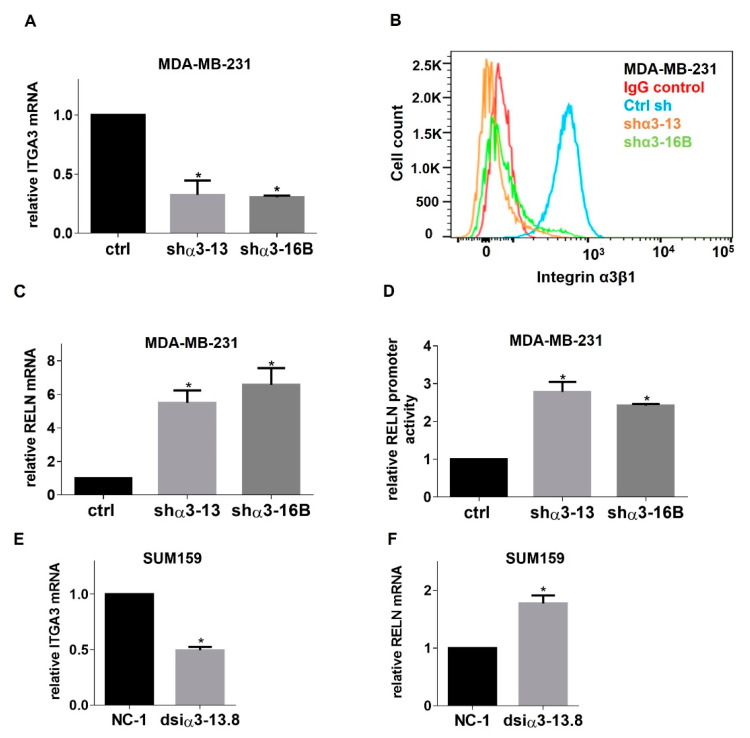
Suppression of integrin α3β1 with shRNA or dicer-substrate siRNA significantly increases RELN mRNA expression in breast cancer cells. (**A**) qRT-PCR was performed to compare ITGA3 mRNA in MDA-MB-231 cells transduced with control shRNA (ctrl) or two distinct α3-targeting shRNAs (shα3-13, shα3-16B). (**B**) Flow cytometry was performed with the anti-α3β1 monoclonal antibody, P1B5, to compare cell surface expression of α3β1 in the cells from (**A**). Control MDA-MB-231 cells were stained with normal mouse IgG as a negative control. (**C**) qRT-PCR was performed to compare RELN mRNA in MDA-MB-231 cells transduced with control or α3-targeting shRNA. (**D**) A dual luciferase reporter assay was performed to compare RELN promoter activity in MDA-MB-231 cells transduced with control or α3-targeting shRNA. (**E**,**F**) qRT-PCR was performed to compare ITGA3 mRNA (**E**) or RELN mRNA (**F**) in SUM159 cells transfected with control dicer-substrate siRNA (NC-1) or α3-targeting dicer siRNA (dsiα3-13.8). Data are average +/− SEM, *n* = 3; * *p* ≤ 0.05. Multiple *t*-test comparison with Sidak–Bonferroni correction (**A**,**C**,**D**); unpaired *t*-test (**E**,**F**).

Our previous study showed that suppression of α3β1 in the TNBC cell line MDA-MB-231, using shRNA to knockdown the α3 integrin subunit (i.e., α3-KD cells), reduced both in vitro invasion and in vivo tumor growth in a xenograft model [14]. Our subsequent gene microarray study revealed an array of genes that were differentially expressed in α3-KD MDA-MB-231 cells, compared with control cells [15]. Among them, expression of the RELN gene was enhanced ~5-fold in α3-KD cells. To confirm α3β1-dependent regulation of RELN, we performed qRT-PCR to compare RELN mRNA levels in MDA-MB-231 cells that were stably transduced with two distinct α3-targeting shRNAs, or with non-targeting shRNA as a control (Figure 1A,B and Figure S1A,B). α3-KD cells showed a significant increase in RELN mRNA compared with control cells (Figure 1C), confirming our previous microarray results.

RELN is epigenetically repressed or silenced in breast cancer [23]. Therefore, we investigated whether expression of α3β1 represses RELN promoter activity. Control or α3-KD MDA-MB-231 cells were transfected with a plasmid containing the luciferase reporter gene under control of a minimal human RELN promoter fragment (−514 to +76 base pairs) [28], and luciferase activity was measured in cell lysates as an indirect measure of RELN promoter activity. We observed significantly higher luciferase signal in the α3-KD cells compared to control cells (Figure 1D), indicating that expression of α3β1 leads to repression of the RELN promoter and suggesting that modulation of this integrin alters RELN gene transcription.

**Figure 2:**
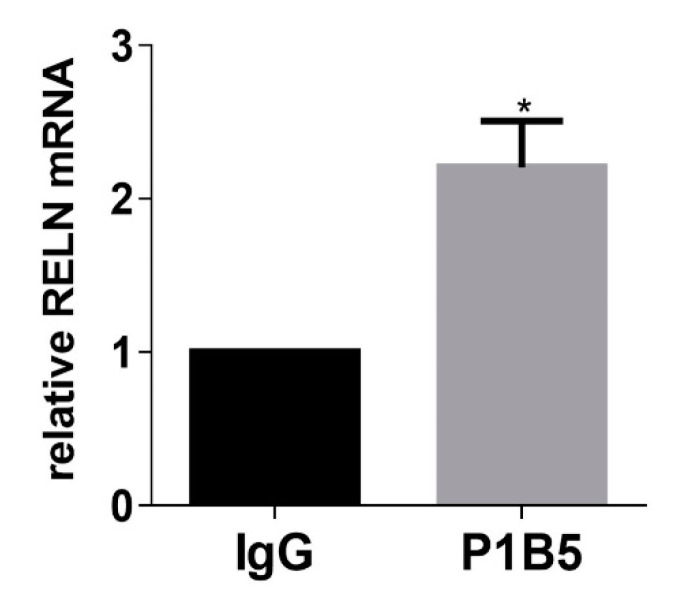
Treatment of MDA-MB-231 cells with a function-blocking antibody against α3β1 increases RELN mRNA expression. qRT-PCR was performed to compare RELN mRNA in MDA-MB-231 cells plated on laminin-332-rich matrix and treated with the α3β1-blocking antibody, P1B5, or with IgG as a control. Data are average +/− SEM, *n* = 3; * *p* ≤ 0.05, unpaired *t*-test.

**Figure 3:**
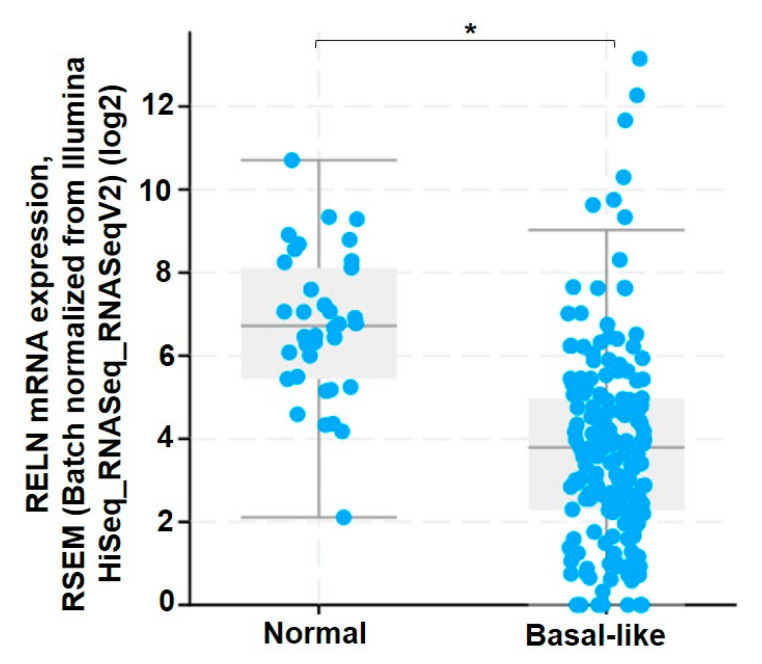
RELN mRNA expression is reduced in basal-like breast cancer. Plot shows RELN gene expression in samples from normal breast tissue versus samples of basal-like subtype of breast cancer, obtained from the Breast Invasion Carcinoma data (TCGA, PanCancer Atlas, cBioPortal); * *p* = 6.77 × 10^−12^ See Materials and Methods for details of analysis.

α3β1-dependent regulation of RELN mRNA was confirmed in a second TNBC cell line, SUM159, by suppressing α3β1 using either α3-targeting siRNA (Figure 1E,F) or a distinct α3-targeting dicer substrate siRNA (Figure S2A,B). A role for α3β1 in the repression of RELN was further confirmed by treatment of MDA-MB-231 cells with the anti-α3β1 monoclonal antibody, P1B5, which blocks α3β1 binding to its laminin ligands [29]. P1B5 treatment similarly led to enhanced RELN mRNA expression (Figure 2). Thus, integrin α3β1 represses RELN gene expression in two different TNBC cell lines. The potential clinical relevance of our findings is supported by our observation that Reelin expression is reduced in basal-like subtypes of breast cancer (which are generally triple-negative), as revealed through analysis of patient data using cBioPortal (Breast Invasive Carcinoma dataset, TCGA, PanCancer Atlas) (Figure 3).

### 2.2. Modulation of RELN Expression in Breast Cancer Cells Alters Invasiveness

**Figure 4:**
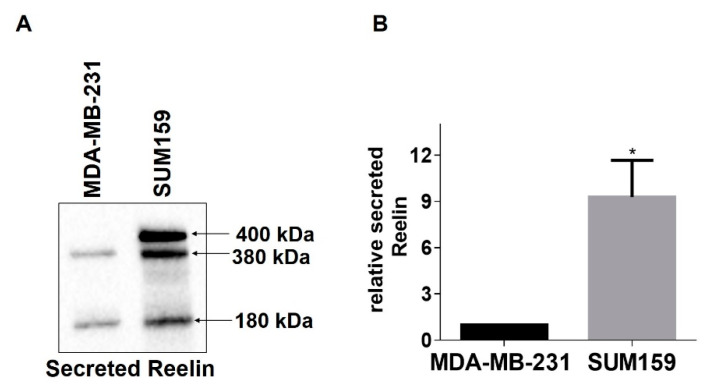
SUM159 cells secrete higher amounts of Reelin protein than MDA-MB-231 cells. (**A**) Representative Western blot of secreted Reelin protein in concentrated conditioned medium collected from MDA-MB-231 or SUM159 cells. Arrows indicate previously described Reelin fragments (400 kDa, 380 kDa, 180 kDa) [30,31]. (**B**) Graph shows quantification of Reelin by Western blot of SUM159 relative to MDA-MB-231 cells. Signals for the different Reelin fragments were combined for each cell line. Data are average +/− SEM, *n* = 3; * *p* ≤ 0.05, unpaired *t*-test.

**Figure 5:**
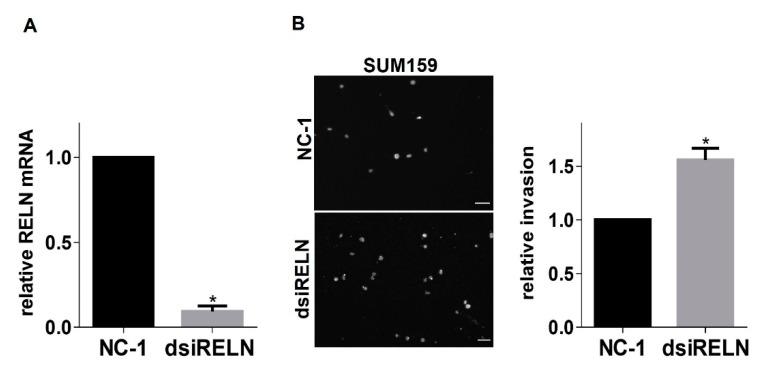
Suppression of RELN using dicer-substrate siRNA increases invasion of SUM159 cells. (**A**) qRT-PCR was performed to compare RELN mRNA expression in SUM159 transfected with control dicer siRNA (NC-1) or dicer siRNA that targets RELN (dsiRELN 13.2). (**B**) Invasive potential of SUM159 cells transfected with NC-1 or dsiRELN 13.2 was compared using Matrigel invasion assays. Images show representative fields of DAPI-stained cells that had invaded to the undersides of transwell filters; scale bar, 100 μM. Graph shows relative cell invasion. Data are average +/− SEM, *n* = 3; * *p* ≤ 0.05, unpaired *t*-test.

**Figure 6:**
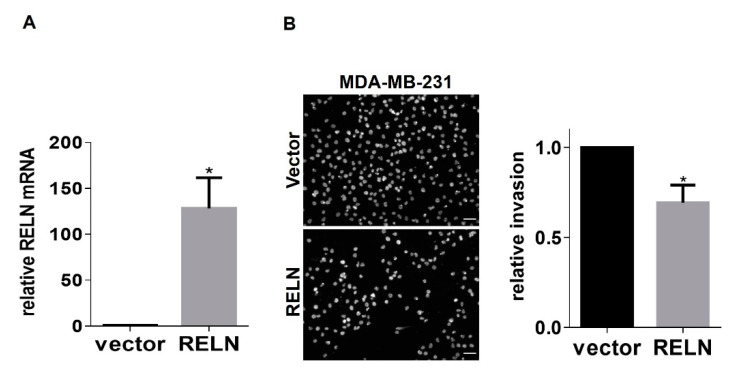
RELN overexpression inhibits invasion of MDA-MB-231 cells. (**A**) qRT-PCR was performed to compare RELN mRNA in MDA-MB-231 cells that overexpress RELN; control, pcDNA3.1 vector. (**B**) Invasive potential of MDA-MB-231 cells transfected with vector or overexpressing RELN was compared using Matrigel invasion assays. Images show representative fields of DAPI-stained cells that had invaded to the undersides of transwell filters; scale bar, 100 μM. Graph shows relative cell invasion. Data are average +/− SEM, *n* = 3; * *p* ≤ 0.05, unpaired *t*-test.

RELN is epigenetically repressed in breast cancer, and low RELN expression correlates with increased cancer cell migration and poor prognosis [23]. Therefore, we next wanted to determine whether genetic modulation of RELN expression alters the invasive properties of breast cancer cells. First, we performed western blots of conditioned culture medium from MDA-MB-231 cells or SUM159 cells to assess relative levels of secreted Reelin. Interestingly, we observed that SUM159 cells secrete ~9-fold higher levels of Reelin than MDA-MB-231 cells (Figure 4A,B), suggesting that SUM159 cells may somehow have escaped RELN repression and providing a potential explanation for why α3 knockdown in these cells produced a more modest induction of RELN (~2-fold increase; see Figure 1F) than we observed in MDA-MB-231 cells (~6- to 7-fold increase; see Figure 1C). Based on these findings, we suppressed RELN in SUM159 cells, or overexpressed it in MDA-MB-231 cells, then assessed effects on cell invasion using Matrigel transwell invasion assays. Knocking down RELN using dicer-substrate siRNA led to a significant increase in SUM159 cell invasion. (Figure 5A,B). Conversely, over-expression of exogenous RELN in MDA-MB-231 cells led to a significant decrease in invasion (Figure 6A,B). Together, these results suggest that higher expression of RELN reduces the invasive potential of TNBC cells.

### 2.3. Reelin that Is Secreted by α3β1-Deficient MDA-MB-231 Cells Inhibits Cell Invasion

**Figure 7:**
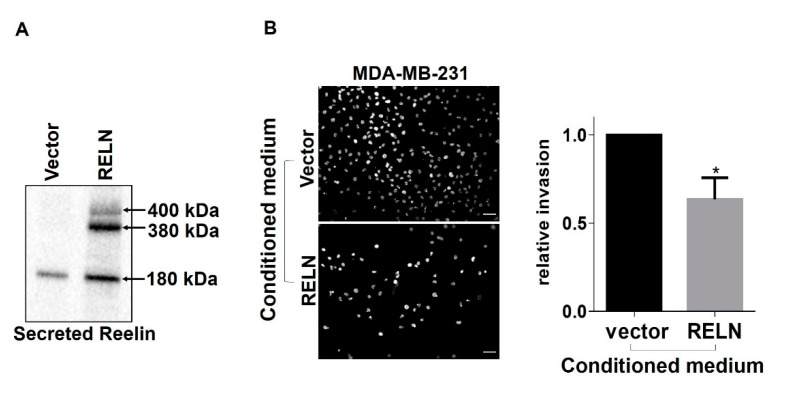
Conditioned medium from MDA-MB-231 cells that overexpress RELN decreases invasion of parental MDA-MB-231 cells. (**A**) Western blot of secreted Reelin in concentrated supernatants of MDA-MB-231 that overexpress RELN; control, pcDNA3.1 vector. Arrows indicate previously described Reelin fragments (400 kDa, 380 kDa, 180 kDa) [30,31]. (**B**) Invasive potential of MDA-MB-231 cells treated with conditioned medium from MDA-MB-231 cells transfected with a RELN over-expression plasmid, or pcDNA3.1 vector as a control, was compared using Matrigel invasion assays. Images show representative fields of DAPI-stained cells that had invaded to the undersides of transwell filters; scale bar, 100 μM. Graph shows relative cell invasion. Data are average +/− SEM, *n* = 3; * *p* ≤ 0.05, unpaired *t*-test.

Consistent with our previous study in which we suppressed α3β1 using shRNA [14], knockdown of α3 in MDA-MB-231 cells using siRNA led to significantly decreased cell invasion (Figure S3A,B). Given our findings that suppression of α3β1 both reduces cell invasion and enhances RELN expression in breast cancer cells, together with reports that Reelin impairs cell invasion in other cancer cell types [23,32], we next wanted to determine whether loss of α3β1-dependent RELN repression, and the resulting increase in secreted Reelin, is sufficient to reduce invasive potential. First, we prepared conditioned culture medium from MDA-MB-231 cells that were stably transduced with a RELN expression plasmid (see Figure 6). As expected, these cells secreted considerably higher levels of Reelin compared with control cells that were transduced with the vector only (Figure 7A). Full length Reelin (410 kDa) is known to undergo proteolytic processing once secreted into the extracellular space [30,31], which leads to larger fragments ranging from 410 kDa to 330 kDa, and a smaller 180 kDa fragment. Importantly, treatment of parental (i.e., α3β1-expressing) MDA-MB-231 cells with conditioned medium from RELN-overexpressing cells led to a modest but significant reduction in invasion, compared with conditioned medium from control cells (Figure 7B). This result is consistent with an inhibitory role for secreted Reelin.

**Figure 8:**
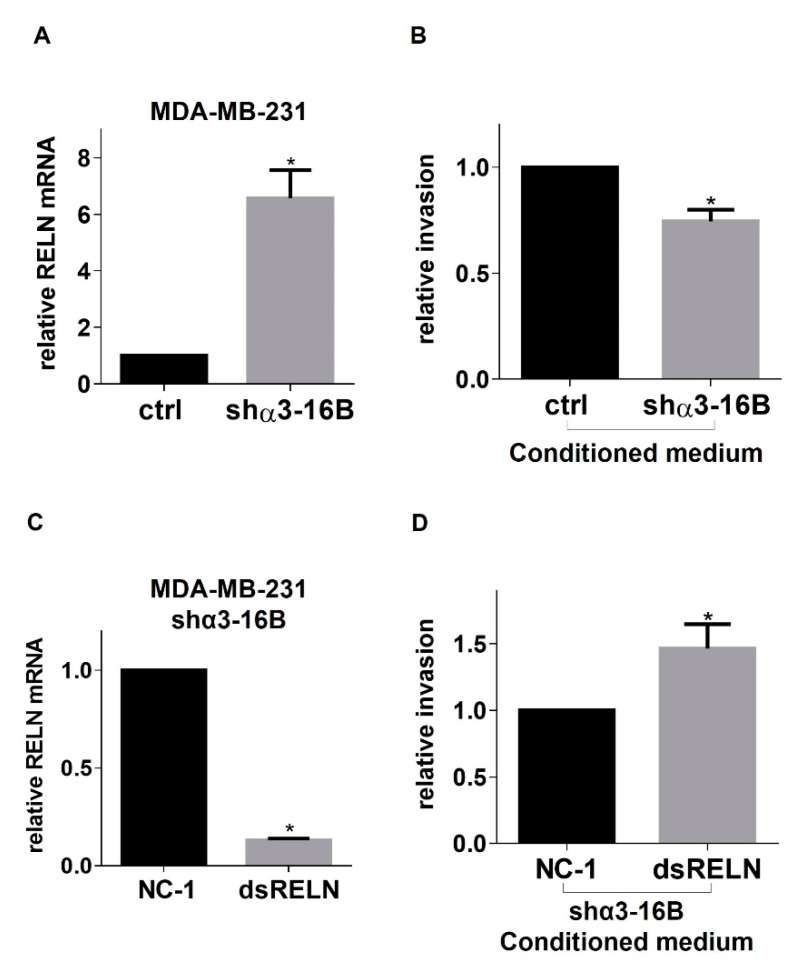
Reelin produced by α3β1-deficient (α3-KD) MDA-MB-231 cells inhibits invasion of parental MDA-MB-231 cells. (**A**) qRT-PCR was performed to compare RELN mRNA in MDA-MB-231 cells stably transduced with control shRNA (ctrl sh) or α3-targeting shRNA (shα3-16B). (**B**) Matrigel invasion assays were performed to compare invasive potential of parental (i.e., α3β1-expressing) MDA-MB-231 cells in the presence of conditioned medium from control (ctrl sh) or α3-KD (shα3-16B) MDA-MB-231 cells. (**C**) qRT-PCR was performed to compare RELN mRNA in α3-KD (shα3-16B) cells transfected with either control (NC-1) or RELN-targeting (dsiRELN) dicer siRNA. (**D**) Matrigel invasion assays of α3-KD (shα3-16B) MDA-MB-231 cells in the presence of conditioned medium from α3-KD (shα3-16B) cells that were transfected with either control (NC-1) or RELN-targeting (dsiRELN) dicer siRNA. Data are average +/− SEM, *n* = 3; * *p* ≤ 0.05, unpaired *t*-test.

**Figure 9:**
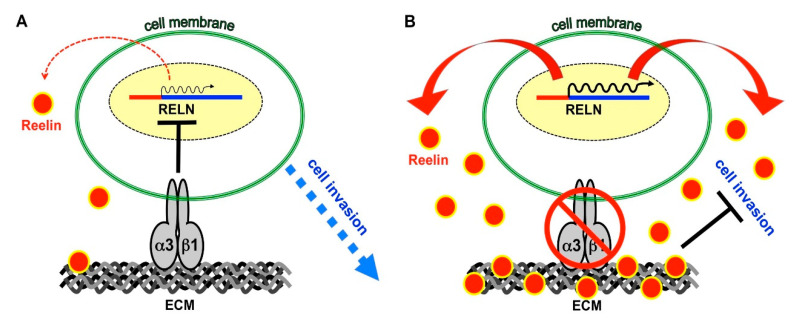
Regulation of the RELN gene by integrin α3β1 modulates breast cancer cell invasion. (**A**) α3β1 represses RELN gene expression by tumor cells, minimizing the production and secretion of Reelin protein (red circles) and supporting cell invasion. (**B**) Suppression or blocking of α3β1 relieves repression of the RELN gene, leading to enhanced secretion of Reelin protein that inhibits cell invasion. ECM, extracellular matrix.

Next, we determined whether the ability of α3β1 to repress Reelin contributes to the pro-invasive properties of this integrin. Invasion of parental MDA-MB-231 cells was modestly but significantly reduced in the presence of conditioned medium collected from α3-KD cells, compared with conditioned medium from control cells (Figure 8A,B). This inhibitory effect on cell invasion was mitigated when RELN was suppressed in α3-KD cells using dicer-substrate siRNA prior to the collection of conditioned medium (Figure 8C,D). Overall, these results indicate that the enhanced expression and secretion of Reelin that occurs in α3-KD cells contributes to the reduced invasive potential of these cells, supporting a model wherein integrin α3β1 represses Reelin expression to promote breast cancer cell invasion (Figure 9A,B).

## 3. Discussion

Numerous studies have demonstrated that integrin α3β1 has pro-tumorigenic/pro-invasive roles in breast cancer cells and other cancer cell types [14,15,33,34,35,36,37]. As reviewed elsewhere [9,38,39], previous studies have established a particularly important role for α3β1 in promoting gene expression programs that control a variety of tumor cell functions, including a secretome that supports a pro-tumorigenic microenvironment. Indeed, we and others have identified individual α3β1-dependent genes that encode secreted proteins, including ECM proteins, extracellular proteases and growth factors, with known roles in modulating the TME [15,40,41,42,43,44,45,46]. The ECM, a major component of the TME of breast cancer, has become the focus of many studies due its significant impact on breast tumorigenesis [5,6]. For example, ECM proteins such as collagens and laminins have been implicated in TNBC, the most aggressive subtype of breast cancer [6,7,10,11]. In the current study, we demonstrated that integrin α3β1, a major receptor for laminins in the ECM, represses the expression of the RELN gene in TNBC cells, thereby identifying a novel, integrin-dependent regulation of tumor cell secretome component that modulates invasive potential. Indeed, RNAi-mediated suppression of α3β1 in TNBC cells led to enhanced RELN mRNA levels and promoter activity and also increased secretion of its protein product, Reelin. Furthermore, our findings establish a causal link between α3β1-dependent repression of RELN and enhanced invasiveness.

Reelin is best known for its role in brain development, where it is critical for regulating neuronal migration, dendrite development and synaptic function [17]. Consequences of RELN gene mutations were first described in the reeler mouse, wherein abnormal Reelin expression caused various abnormalities in the brain including deficits in neuronal positioning and cortical lamination [47,48,49]. In humans, altered Reelin expression is caused mainly by mutations in the RELN gene or hypermethylation of the RELN promoter, and it has been associated with various brain disorders such as Alzheimer’s disease, schizophrenia and depression [50]. More recent studies have described Reelin expression in non-neuronal tissues such as liver and kidney, as well as in normal breast tissue where it is necessary for mammary gland development [51]. Moreover, several studies have now shown that Reelin expression is reduced in many cancers, including breast cancer [23,24,25]. Interestingly, Reelin is differentially expressed within different breast cancer subtypes; Her2-positive breast tumors were found to express higher levels of Reelin compared to TNBC tumors and metastases [26,27]. Our bioinformatic analysis using the Breast Invasive Carcinoma dataset in cBioPortal (Figure 3), further reveals that RELN mRNA expression is reduced in basal-type tumor samples compared to normal tissue samples. Although reduced Reelin expression in breast cancer has been associated with hypermethylation of the RELN gene, disease progression, and decreased survival [23], mechanisms through which the RELN gene is regulated in breast cancer remain unknown. The current study identifies a novel role for integrin α3β1 in repressing Reelin expression, adding to a number of other important functions that this integrin plays in promoting breast cancer [9].

Previous studies from our group and others indicate that the majority of α3β1-dependent secretome genes are up-regulated in tumor cells or immortalized cell lines by the expression of α3β1, and many of these genes have been linked to pro-tumorigenic or pro-angiogenic functions, as reviewed in [52]. Therefore, it is noteworthy that our current study identifies a role for α3β1 in the down-regulation of the RELN gene, as Reelin is an extracellular protein that inhibits breast cancer cell invasion. Collectively, these findings suggest that pro-invasive effects of integrin α3β1 stem from its ability to both up-regulate genes that promote invasion and down-regulate genes that inhibit invasion. The gene regulatory functions of α3β1 in tumor cells reinforce the concept that this integrin has central roles in cancer that extend beyond its ability to directly control cell adhesion and motility following its binding to laminins in the ECM [39].

Some α3β1 functions may require its ability to crosstalk with growth factors or cytokines. Indeed, it has been known for more than three decades that many integrins can modulate intracellular signaling pathways through crosstalk with receptors for growth factors or cytokines, and the importance of mechanical properties of the extracellular microenvironment in this regulation has become clear, as reviewed elsewhere [53]. One of the most intensively studied examples is crosstalk between certain integrins and TGF-β signaling, which can occur when integrins regulate the expression of TGF-β signaling components, physically interact with TGF-β receptors, or modulate the functions of downstream signaling effectors [54,55]. Interestingly, TGF-β signaling has been inversely linked to RELN expression in cancer cells. Indeed, studies in hepatocellular or esophageal carcinoma cells have shown that enhanced cell migration in response to TGF-β1 is linked to the repression of RELN gene expression [56,57,58]. Numerous studies have linked certain α3β1 functions to TGF-β signaling, as reviewed [54], suggesting possible co-involvement of α3β1 and TGF-β in the repression of RELN expression. Moreover, we recently showed that α3β1 regulates the secretion by tumor keratinocytes of not only ECM proteins and extracellular proteases, but also of some growth factors and cytokines [59], leaving open the possibility that integrin crosstalk with other growth factor/cytokine receptors may regulate Reelin expression or function.

Our current findings enhance our understanding of how tumor cell α3β1 contributes to cancer progression through control of the secretome and subsequent modulation of the tumor microenvironment [38]. Future studies to investigate the mechanisms through which α3β1 represses RELN gene expression should offer new insights into therapeutic strategies to block this regulation in breast cancer cells and promote up-regulation of anti-invasive proteins such as Reelin. Moreover, it is significant that α3β1-dependent modulation of the tumor microenvironment includes both upregulation of pro-cancer genes and downregulation of anti-cancer genes, as it suggests that targeting α3β1 therapeutically may have the pleiotropic effect of targeting multiple genes that impact disease progression. Future studies will investigate the extent to which α3β1 regulates different gene targets through common transcriptional or post-transcriptional pathways.

## 4. Materials and Methods

### 4.1. Cell Culture

Control and α3-KD MDA-MB-231 cells (American Type Culture Collection, ATCC, Manassas, VA, USA) were described previously [14] and authenticated by STR-profiling (ATCC). MDA-MB-231 cell variants were cultured in Dulbecco’s Modified Eagle Medium (DMEM) (Corning, Waltham, MA, USA) supplemented with 10% fetal bovine serum (FBS) (Gemini Bio-Products, West Sacramento, CA, USA) and 1% L-glutamine (Gibco, Waltham, MA, USA). SUM159 cells (Asterand) were cultured in Ham’s F12 media (Gibco) supplemented with 5% fetal bovine serum, 5 µg/mL insulin (Sigma, St. Louis, MO, USA), 1 µg/mL hydrocortisone (Sigma) and L-glutamine (Gibco). All cells were maintained at 37 °C, 5% CO_2_.

### 4.2. siRNA- and shRNA-Mediated Gene Suppression

MISSION lentiviral short-hairpin RNA (shRNA) constructs (Sigma, St. Louis, MO, USA) were used to target the human *ITGA3* gene (shRNA 13, 16B), and a non-targeting shRNA was used as control (Sigma), as described previously [14]. To suppress α3 using siRNA, cells were transfected for 72 h with siRNA that targets α3 (sia3-78, 59-GUGUACAUCUAUCACAGUA-39; Sigma-Aldrich, St. Louis, MO, USA) or luciferase as a control (Dharmacon™, Lafayette, CO, USA) using lipofectamine^TM^ 2000 (Invitrogen, Waltham, MA, USA) diluted in Opti-MEM (Gibco) according to the manufacturer’s instructions. For the suppression of α3 using dicer-substrate siRNA; cells were transfected using RNAiMax and a dicer-substrate non-targeting control (IDT, Coralville, IA, USA) or a dicer-substrate targeting α3 (hs.Ri.ITGA3.13.8, IDT). RELN gene suppression was achieved by transfecting cells for 72 h with RNAiMax and a pre-designed dicer-substrate siRNA (DsiRNA) that targets the human RELN gene (hs.Ri.Reln13.2; Integrated DNA Technologies, IDT, Coralville, Iowa). Non-targeting DsiRNA (NC-1) from IDT was used as a negative control.

### 4.3. Stable Overexpression of Recombinant RELN

MDA-MB-231 cells were transfected with a RELN expression vector, pCrl, a gift from Tom Curran (Addgene plasmid # 122443; http://n2t.net/addgene:122443; RRID: Addgene_122443) and first cloned by D’Arcangelo et al. [16]. Cells were transfected at 80% confluence with 2.5 μg of unlinearized pCrl plasmid DNA or empty backbone (pcDNA3, neomycin-resistant), using Lipofectamine LTX (Invitrogen, Waltham, MA) for 24 h. Stable transfectants were selected by growing cells in 600 μg/mL Geneticin (G418) (Gibco). Levels of Reelin mRNA and protein were assessed by qRT-PCR and western blot, respectively.

### 4.4. Real-Time Quantitative PCR (qRT-PCR)

RNA was isolated from cells using Trizol reagent (Invitrogen Corp., Carlsbad, CA, USA) followed by Qiagen RNeasy assay (Qiagen, Hilden, Germany) according to the manufacturer’s protocol. Isolated RNA was treated with DNAse (Turbo DNA-free kit, Ambion, Waltham, MA, USA), then cDNA was synthesized using iScript™ cDNA Synthesis Kit (Bio-Rad, Hercules, CA, USA). qRT-PCR was performed using SYBR Green (SsoAdvanced™ Universal SYBR^®^ Green Supermix, Bio-Rad) on a Bio-Rad CFX96 Touch thermocycler (Bio-Rad). Amplification conditions were as follows: 95 °C, 3 min; (95 °C, 10 s; 55 °C 30 s) for 39 cycles. A Bio-Rad CFX Manager software was used to acquire Ct values. Primers were as follows: ITGA3, forward 5′-GCAGGTAATCCATGGAGAGAAG-3′, reverse 5′-CCACTAGAAGGTCTGGGTAGAA-3′; RELN, forward 5′-TGCTGGAATACACTAAGGATGC-3′, reverse 5′-GAAGGCACTGGGTCTGTACG-3′; PUM1, forward TACGTGGTCCAGAAGATGATTG, reverse GCCATAGGTGTACTTACGAAGAG; PSMC4, forward GGAGGTTGACTTGGAAGACTATG, reverse GACAGCCAACATTCCACTCT. qRT-PCR signals for all test genes were normalized to the average of those for PUM1 and PSMC4.

### 4.5. Western Blot

To measure secreted reelin levels, 3 × 10^5^ cells were seeded onto 6-well plates and cultured to ~70% confluency, then growth medium was replaced with serum-free medium. After 24 or 48 h, supernatants were collected and concentrated using Amicon Ultra-15 centrifugal filters (Merck Millipore, Tullagreen, Carrigtwohill, Co Cork, Ireland). Cell lysates were obtained by adding cell lysis buffer (Cell Signaling, Waltham, MA, USA) supplemented with protease inhibitor (Roche, St. Louis, MO, USA) to the adherent cells. A BCA protein assay kit (Thermo Scientific, Rockford, IL, USA) was used to measure protein concentration in cell lysates or concentrated supernatants, and equal protein (20 µg) was subject to Western blot. For Reelin blots: Samples were run under reducing conditions using gradient gels (NuPAGE^TM^ 4–12% Bis-Tris gels, Invitrogen, Carlsbad, CA, USA). For α3 and ERK2 blots, samples were run under non-reducing conditions using homemade 10% SDS-PAGE gels. All samples were transferred to nitrocellulose membranes, blocked in 5% bovine serum albumin, and incubated with primary antibodies. The following primary antibodies were applied overnight at 4 °C: Anti-integrin α3 (1: 1000 dilution) [60]; anti-ERK2 (1:1000 dilution; Santa-Cruz, Dallas, TX, USA); anti-Reelin (1:500 dilution; clone 142, Sigma). Secondary antibodies used were goat anti-rabbit (1:10,000 dilution, Cell Signaling, Waltham, MA, USA), or goat anti-mouse HRP (Thermo Fisher, Waltham, MA, USA). Blots were developed using Clarity Western ECL substrate (Bio-rad, Hercules, CA, USA) and imaged using the Bio-rad ChemiDOC^TM^ MP Imaging system. ImageJ was used to quantify western blot signals. The uncropped western blotting figures can be found in Figure S4.

### 4.6. RELN Promoter Transfection and Luciferase Assay

The luciferase reporter plasmid (RELN-514) containing a minimal promoter of the human RELN gene (−514 to +76 base pairs relative to the transcription start site) was a generous gift from Dr. D. R. Grayson (Department of Psychiatry, University of Illinois at Chicago, Chicago, IL, USA) [28]. Cells were plated on 12-well plates and grown to ~80% confluence before being transfected with the RELN promoter construct, or with empty vector (pGL3-Basic) as control using lipofectamine 2000 (Invitrogen). A Renilla gene reporter plasmid was co-transfected as an internal control for transfection efficiency [61]. Thirty-six hours post-transfection, luciferase activity was measured in triplicate wells using a SpectraMaxi i3 Multi-Mode Platform plate reader (Molecular Devices) and a dual luciferase assay kit (Promega, Madison, WI, USA) according to manufacturer’s instructions.

### 4.7. Matrigel Invasion Assay

8 × 10^5^ cells were seeded in complete growth medium onto 24-well transwell inserts (8 µM filters, Corning, Waltham, MA, USA) coated with growth factor-reduced Matrigel (Fisher Scientific, Waltham, MA). Serum concentration was increased in the lower chambers as chemoattractant (MDA-MB-231, 20% FBS; SUM159, 10% FBS). For Matrigel invasion experiments involving conditioned serum-free medium, 10% FBS was added in the lower chambers as chemoattractant. Plates were incubated at 37 °C for 18 h to allow cells to invade through the Matrigel layer, and cotton swabs were used to remove non-invading cells from the top sides of filters. The bottom sides of filters were then fixed with 100% ice-cold methanol and stained with DAPI. Cells were counted from 3 random 10X fields using a Nikon eclipse TE2000-U inverted microscope. Cell invasion was quantified from 3 independent experiments, wherein each condition was plated in duplicate.

### 4.8. Flow Cytometry

MDA-MB-231 cells were trypsinized and resuspended in blocking buffer (PBS/10% goat serum), then incubated on ice for 15 min. Cells were washed with 0.1% BSA/PBS, pelleted, then resuspended in 5 µg/mL of either primary antibody against integrin α3 (EMD Millipore Corp, Billerica, MA, USA) or normal mouse IgG (Santa Cruz Biotechnology, Dallas, TX, USA) on ice for 45 mins. Cells were washed and incubated with secondary antibody (allophycocyanin, crosslinked, goat anti-mouse IgG, Invitrogen) at 1:200 dilution on ice for 45 min, then fixed in 2% formaldehyde/PBS for 10 min on ice. Surface integrin α3 levels were measured using the FACSCalibur (Becton Dickinson, Franklin Lakes, NJ, USA); analysis was done with FlowJo software.

### 4.9. Preparation of Laminin-332-Rich ECM from SCC-25 Cells

Human squamous cell carcinoma cells (SCC-25) [62] were used to prepare laminin-332-rich ECM, as we described previously [46]. Briefly, SCC-25 cells were grown on 12-well tissue culture plates in DMEM:HAM’s F-12 medium supplemented with 10% FBS and 0.4 µg/mL hydrocortisone. Once confluence was reached, cells were removed using TryPLE (Gibco, Denmark). The laminin-332-rich ECM left on the plates was then treated with 0.5 mg/mL soybean trypsin inhibitor (Gibco, Denmark) in PBS and blocked with 1 mg/mL BSA (Sigma) in PBS.

### 4.10. Treatment of Cells with Anti-α3β1 Blocking Antibody

2 × 10^5^ MDA-MB-231 cells were suspended in serum-free medium containing 10 µg/mL mouse IgG (Santa Cruz Biotechnology) or the anti-α3β1 monoclonal antibody, P1B5 (EMD Millipore) for 30 min, then replated onto laminin-332-rich ECM prepared as described above. RNA was harvested and qRT-PCR performed as described above to measure RELN mRNA.

### 4.11. Bioinformatics

To compare RELN mRNA expression between normal and basal-like breast cancer samples, we used the Breast Invasive Carcinoma data set (TCGA, PanCancer Atlas: https://www.cancer.gov/tcga) available in cBioPortal. Samples were grouped into normal breast tissue or basal-like subtype of breast cancer, and RELN gene expression was compared between these two sample sets.

### 4.12. Statistical Analyses

Unpaired (two-tailed) *t*-test was used for experiments involving the comparison of two groups. Multiple *t*-test comparison with Sidak–Bonferroni correction was performed for experiments involving more than two groups. All data are average +/− SEM of *n* = 3. Graphs were generated and statistical analyses performed using GraphPad Prism. Statistical significance was annotated as follows: n.s. (not significant), *p* > 0.05; * *p* ≤ 0.05.

## 5. Conclusions

Integrins are essential for the maintenance of cellular homeostasis in the normal cell. However, integrins are also highly expressed in breast cancer cells where they drive invasion and tumor growth. In order to fully exploit the potential of integrins as therapeutic targets for breast cancer, it is crucial to have a more complete understanding of the mechanisms by which these cellular receptors promote breast cancer invasion.

Our current findings demonstrate that integrin α3β1 significantly represses the expression of Reelin, a secreted glycoprotein that is known to be repressed or silenced in breast cancer. Because loss of Reelin has been associated with decreased survival of breast cancer patients, we sought to determine the effects of α3β1-mediated silencing of Reelin on breast cancer cell invasion. Our findings demonstrate a critical role for Reelin in breast cancer cell invasion, as increasing Reelin expression in Reelin-low cells decreased invasion, while decreasing Reelin expression in Reelin-high cells increased invasion. We further showed that treating α3β1-expressing MDA-MB-231 cells with conditioned medium from α3-deficient cells wherein RELN was blocked led to increased invasion. Overall, our study identifies an important role for α3β1-dependent regulation of RELN that impacts breast cancer cell invasion, highlighting the potential clinical importance of exploiting integrin α3β1 as a therapeutic target for breast cancer.

## Data Availability

Breast Invasive Carcinoma data set is available through cBioPortal (TCGA, PanCancer Atlas: https://www.cancer.gov/tcga).

## Supplementary Materials

The following are available online at https://www.mdpi.com/2072-6694/13/2/344/s1, Figure S1: Measurement of α3 protein in α3-expressing and α3-deficient MDA-MB-231 cells by Western blot, Figure S2: Measurement of RELN mRNA in SUM159 cells upon siRNA-mediated knockdown of ITGA3, Figure S3: Invasion of MDA-MB-231 cells is reduced upon siRNA-mediated knockdown of ITGA3, Figure S4: Uncropped western blotting figures.
