# Supplementary material for: Integrin α3β1 Represses Reelin Expression in Breast Cancer Cells to Promote Invasion
Source: Cancers (Basel). 2021 Jan 19;13(2):344. doi: 10.3390/cancers13020344 (PMC7832892; doi:10.3390/cancers13020344)
Supplement: Supplementary file 1 [file cancers-13-00344-s001.zip › cancers-1062590-supplement.pdf]

cancers

MDPI

Supplementary Materials

Integrin α3β1 Represses Reelin Expression in Breast Cancer Cells to Promote Invasion

Abibatou Ndoye, Rakshitha Pandulal Miskin and C. Michael DiPersio

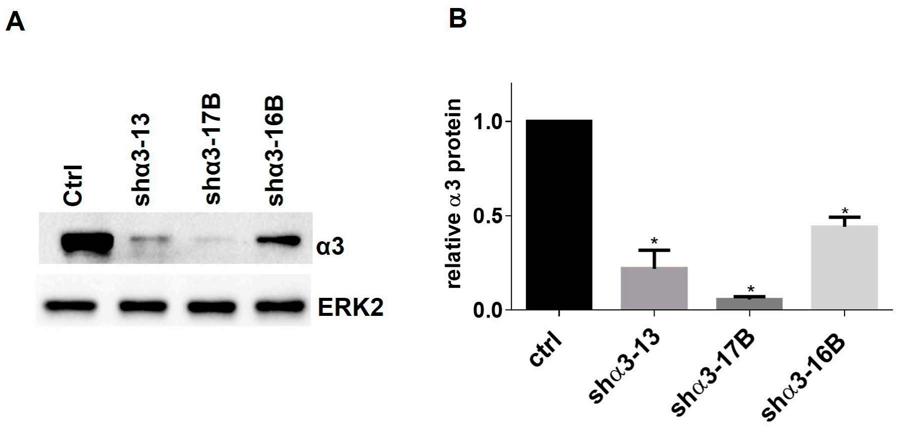

Figure S1. Measurement of α3 protein in α3-expressing and α3-deficient MDA-MB-231 cells. **A**. Representative western blot (non-reducing) of α3 in MDA-MB-231 cells transduced with control shRNA (ctrl) or three distinct α3-targeting shRNAs (shα3-13, shα3-17B, shα3-16B); control, ERK2. **B**. Graph shows the quantification of α3 protein; data normalized to ERK protein levels. Data are average +/- SEM, n=3; *$p \leq 0.05$; multiple t-test comparison with Sidak-Bonferroni correction.

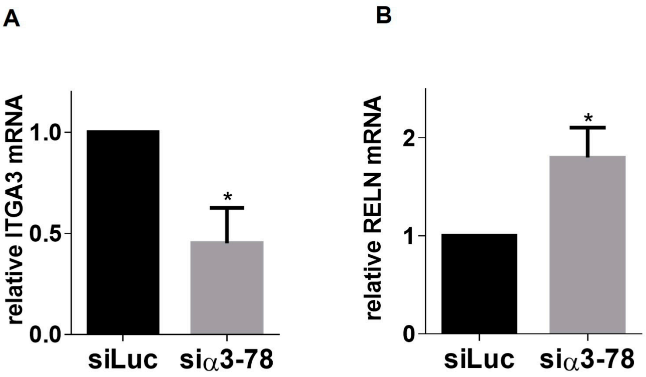

Figure S2. Measurement of RELN mRNA in SUM159 cells upon siRNA-mediated knockdown of ITGA3. **A, B**. qRT-PCR was performed to compare ITGA3 mRNA (A) or RELN mRNA (B) in SUM159 cells transfected with control siRNA (siLuc) or siRNA that targets α3 mRNA (siα3 -78). Data are average +/-SEM, n=3; *$p \leq 0.05$, unpaired t-test.

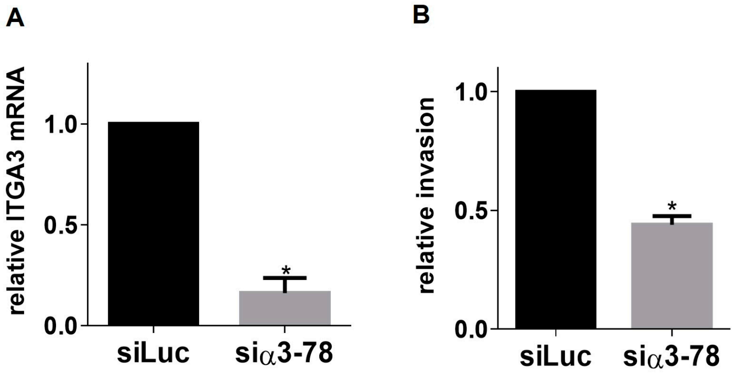

Figure S3. Invasion of MDA-MB-231 cells is reduced upon siRNA-mediated knockdown of ITGA3. **A**. qRT-PCR was performed to compare ITGA3 mRNA in MDA-MB-231 cells transfected with control siRNA (siLuc) or α3-targeting siRNA (siα3 -78). **B**. Invasive potential of MDA-MB-231 cells transfected with siLuc or siα3-78 cells was compared using Matrigel invasion assays. Graph shows relative cell invasion. Data are average +/- SEM, n=3; *$p \leq 0.05$, unpaired t-test.
