# Supplementary figures and images for: Integrin α3β1 Represses Reelin Expression in Breast Cancer Cells to Promote Invasion
Source: Cancers (Basel). 2021 Jan 19;13(2):344. doi: 10.3390/cancers13020344 (PMC7832892; doi:10.3390/cancers13020344)

## Figure S4: Uncropped western blotting figures

Figure S1A

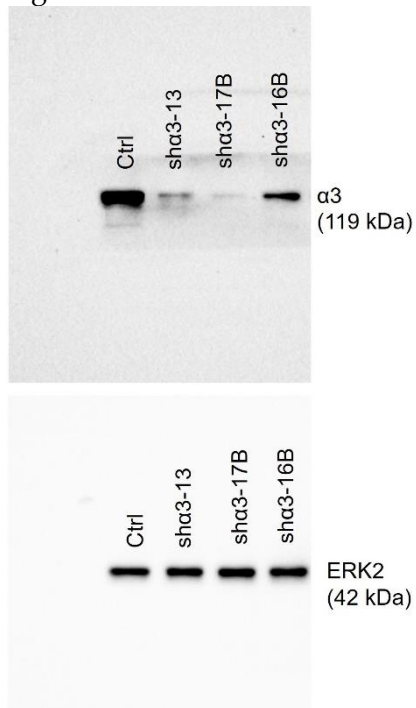

Figure 3A

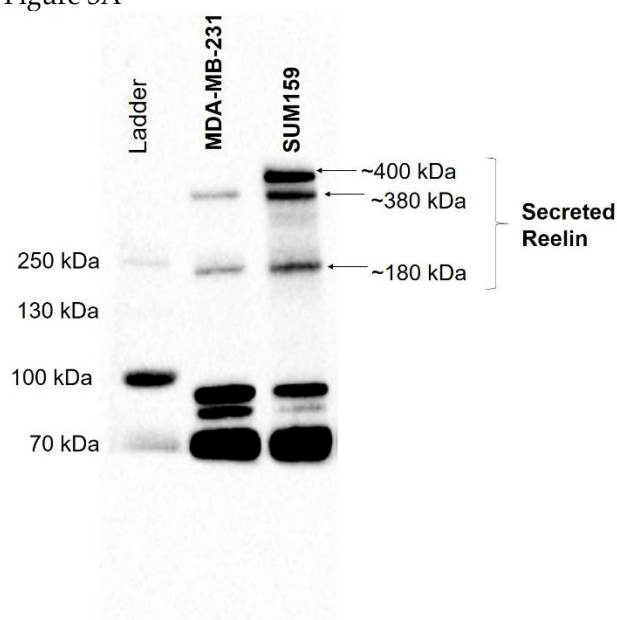

Figure 6A

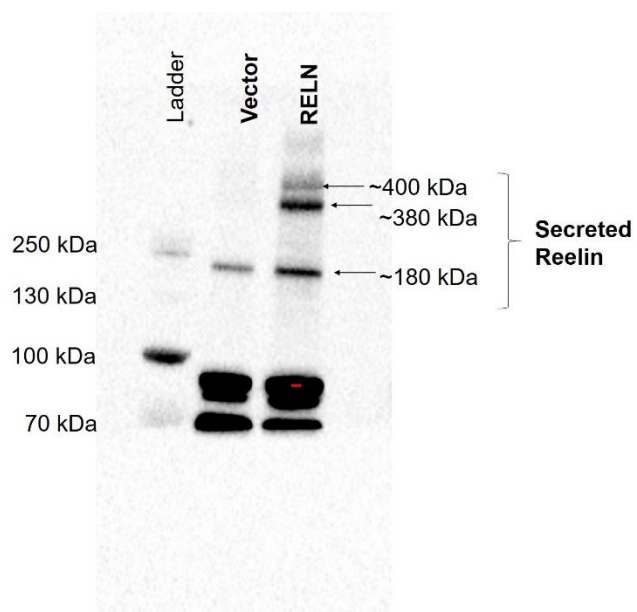

Supplement: Supplementary file 1 [file cancers-13-00344-s001.zip › Figure S4 - Uncropped Western Blotting Figures.pdf]
